# Protective Effects of L-Cysteine Against Cisplatin-Induced Oxidative Stress-Mediated Reproductive Damage

**DOI:** 10.3390/antiox13121443

**Published:** 2024-11-23

**Authors:** Yi-Fen Chiang, Yi-Tzu Chen, Ko-Chieh Huang, Wei-Lun Hung, Cheng-Pei Chung, Tzong-Ming Shieh, Yun-Ju Huang, Mohamed Ali, Shih-Min Hsia

**Affiliations:** 1 School of Nutrition and Health Sciences, College of Nutrition, Taipei Medical University, Taipei 11031, Taiwan; 2 School of Food Safety, College of Nutrition, Taipei Medical University, Taipei 11031, Taiwan; 3 Department of Nutrition and Health Sciences, Chang Gung University of Science and Technology, Taoyuan 33303, Taiwan; 4 School of Dentistry, College of Dentistry, China Medical University, Taichung 40402, Taiwan; 5 Department of Biotechnology and Food Technology, Southern Taiwan University of Science and Technology, Tainan 710301, Taiwan; 6 Clinical Pharmacy Department, Faculty of Pharmacy, Ain Shams University, Cairo 11566, Egypt; 7 Department of Obstetrics and Gynecology, University of Chicago, Chicago, IL 60637, USA; 8 Graduate Institute of Metabolism and Obesity Sciences, College of Nutrition, Taipei Medical University, Taipei 11031, Taiwan; 9 Nutrition Research Center, Taipei Medical University Hospital, Taipei 11031, Taiwan; 10 TMU Research Center for Digestive Medicine, Taipei Medical University, Taipei 110301, Taiwan

**Keywords:** cisplatin, L-cysteine, male reproductive damage, oxidative stress

## Abstract

Cisplatin (CIS) is a widely used chemotherapeutic agent, but its side effects, such as oxidative stress, inflammation, and apoptosis, often lead to male reproductive damage. Oxidative stress, primarily caused by the excessive generation of reactive oxygen species (ROS), plays a critical role in disrupting testicular homeostasis, resulting in spermatogenic impairment and tissue injury. L-cysteine (CYS), a semi-essential amino acid with potent antioxidant and anti-inflammatory properties, may offer protection against CIS-induced oxidative damage. This study aimed to assess the protective potential of CYS against CIS-induced male reproductive toxicity using in vivo and in vitro models. In vitro, treatment of TM3 (Leydig) and TM4 (Sertoli) cells with CIS led to increased ROS levels, reduced cell viability, and elevated apoptosis and inflammation, all of which were significantly ameliorated by subsequent CYS exposure. In vivo, CIS-treated male rats displayed heightened oxidative stress, impaired spermatogenesis, and histopathological damage in reproductive organs. However, CYS administration for 21 days significantly reduced oxidative stress, improved sperm viability, and protected testicular tissues from damage. These findings suggest that CYS has a protective effect against CIS-induced oxidative stress and male reproductive damage, making it a promising therapeutic agent for mitigating CIS-induced reproductive toxicity.

## 1. Introduction

According to the World Health Organization (WHO) and American Medical Association (AMA) definitions of infertility, it is defined by the failure to conceive after more than 12 months of regular unprotected sexual intercourse [1]. Approximately 48.5 million couples have this problem and 20–30% of male infertility contributes to 50% of cases around the world [2]. The testis plays a crucial role in the male reproductive system, and several studies have suggested that a wide variety of endogenous and exogenous factors can disrupt male reproductive function. Approximately 70–80% of cases are caused by testicular defects, including chromosomal/genetic abnormalities (e.g., Klinefelter syndrome), cryptorchidism, varicocele, orchitis, cancer treatments (chemotherapy/radiotherapy), environmental factors (such as environmental toxins, high temperature, radiation, and smoking), autoimmune diseases (antisperm antibodies), and testicular torsion and trauma [3].

Several studies have suggested that oxidative stress and apoptosis are key mediators of male infertility by causing sperm dysfunction [4]. Oxidative stress is a disturbance in the balance between the production of reactive oxygen species (free radicals) and antioxidant defenses. Cisplatin (CIS), or cis-diamminedichloroplatinum (II), is an effective anticancer chemotherapy agent widely used to treat various cancer types. However, cisplatin is often accompanied by numerous toxicities as it reacts with the N7 reactive center of DNA, leading to DNA–DNA crosslinks and the inhibition of DNA replication or transcription [5].

Subsequently, CIS causes reproductive toxicity, resulting in the production of free reactive oxygen species (ROS), pro-inflammatory cytokines, mitochondrial dysfunction, and apoptosis. The testis is the target of chemotherapeutic agents due to spermatogenesis and spermiogenesis, in which testicular cell division and morphological changes occur [6,7,8].

Leydig cells are interstitial cells located adjacent to the seminiferous tubules in the testis [9].The mature function of Leydig cells is mainly to produce testosterone under the control of pituitary luteinizing hormone (LH) by action on steroidogenesis. Sertoli cells are the epithelial supporting cells of the seminiferous tubules. Sertoli cells are mainly regulators of spermatogenesis in the adult testis and express functional receptors for follicle-stimulating hormone (FSH) [10,11]. They support fetal germ cell changing in the adult male pathway and are essential for germ cell development. CIS causes Leydig cell dysfunction, leading to impaired testosterone secretion and disruption of the Sertoli cells in seminiferous epithelium, which results in the impairment of spermatogenesis. In addition, cisplatin induces epididymal toxicity by decreasing epididymal sperm count [12,13].

Because the concomitant use of antioxidants with CIS may reduce the efficacy of cytotoxic drugs, it is essential to identify a potential substance specifically for the treatment of testicular damage. The semi-essential amino acid L-cysteine (CYS), which contains a thiol side chain that modulates enzymatic reactions, plays diverse roles within cells, including maintaining antioxidant homeostasis [14]. It is naturally found in meat, fish, grains, dairy, soybeans, and eggs [15,16]. CYS serves as a precursor to inorganic sulfates, coenzyme A, and glutathione [17]. Its primary functions include antioxidation and contributing to the stability of structural proteins [18].

Therefore, this study aimed to investigate the protective effects of CYS on CIS-induced testicular damage through comprehensive in vivo and in vitro experiments. We assess the potential of CYS to mitigate oxidative stress, reduce apoptosis, and preserve the structural integrity and function of testicular cells in the presence of CIS. We explore the mechanisms by which CYS may counteract the adverse effects of CIS on male reproductive health.

## 2. Materials and Methods

### 2.1. Chemicals, Reagents, and Antibodies

The materials used include Dulbecco’s modified Eagle’s medium (DMEM)/Ham’s F-12 (12500062, Gibco (Grand Island, NY, USA)), Penicillin streptomycin solution, 100× (30-002-CI, CORNING (Manassas, VA, USA)), fetal bovine serum (FBS, MT-35-010-CV, CORNING), Horse serum (HS, 16050114, Thermo fisher (Waltham, MA, USA)), vitamin C (VITC, A4544, Sigma (St. Louis, MO, USA)), gallic acid (GA, G7384, Sigma), L-cysteine (CYS, 168149, Sigma), cisplatin (CIS, 13119, Cayman (Ann Arbor, MI, USA)), a 2′, 7′-dichlorodihydrofluorescein diacetate (DCFDA, 601520, Cayman), 3-(4,5-dimethylthiazol-2-yl)-5-(3-carboxymethoxyphenyl)-2-(4-sulfophenyl)-2H-tetrazolium (MTS) assay kit (ab197010, Abcam (Cambridge, MA, USA)), and a 2,2-Diphenyl-1-Picrylhydrazyl (DPPH) Commercial kit (D678, Dojindo, Kumamoto, Japan).

### 2.2. Experimental Cell Model

The TM3 mouse Leydig cell line and the TM4 mouse Sertoli cell line were used as experimental models and were obtained from the American Type Culture Collection (ATCC, Manassas, VA, USA). Cells were maintained within passages 3 to 15. The TM3 and TM4 cells were cultured in Dulbecco’s modified Eagle’s medium/Ham’s F-12 supplemented with 5% horse serum, 2.5% fetal bovine serum, 100 μg/mL streptomycin, and 100 U/mL penicillin at 37 °C in an atmosphere containing 5% CO_2_. The cells were passaged every 3 days once they grew to approximately 90% confluence.

### 2.3. Cell Viability Analysis

The cytotoxicity of CIS and CYS was determined by MTS. TM3 and TM4 cells were, respectively, seeded in 96-well plates at a density of 5 × 10^3^ and 3 × 10^3^ cells per well for 48 h. TM3 and TM4 cells were co-treated with different concentrations of CYS (0.1, 0.2, 0.5, 1, and 2 mM) or CIS (5, 7.5, 10, 15, and 20 µM) for 24 or 16 h. After the treatment, 20 µL MTS was added, followed by incubation at 37 °C for 2.5 h protected from light. The absorbance of each well was measured using a VersaMax microplate reader (Molecular Devices, San Jose, CA, USA) at a wavelength of 490 nm.

The cell morphology and survival following CIS and CYS treatments were determined using crystal violet staining. TM3 and TM4 cells were seeded in 12-well plates at a density of 4 × 10^4^ cell per well for 48 h. The cells were then co-treated with varying concentrations of CYS (0.1, 0.2, 0.5, and 1 mM) and induced with CIS (7.5 or 10 µM) for 24 and 16 h, respectively. After treatment, the cells were fixed in methanol and stained with 0.01% crystal violet at room temperature for 20 min. The excess dye was removed, and the stained cells were photographed. The dye was then dissolved in DMSO with shaking until completely dissolved. The absorbance of each well was measured using a VersaMax microplate reader at a wavelength of 570 nm.

### 2.4. Testosterone Evaluation in the Culture Medium

After treatment, the TM3 cell supernatant was collected, and testosterone concentration was measured using a testosterone ELISA kit (582701, Cayman) according to the manufacturer’s instructions. The concentration was normalized to the cell count.

### 2.5. 2,2-Diphenyl-1-Picrylhydrazyl (DPPH) Assay

The scavenging activity of CYS was determined by a DPPH Commercial Kit. The different concentrations of CYS (20, 30, 50, 75, and 150 μg/mL) were mixed with 100 μL DPPH solution in 96-well plates and then incubated at room temperature in the dark for 30 min. The absorbance of each well was measured using a VersaMax microplate reader at the wavelength of 517 nm and using the following formula:

Scavenging activity (%) = [control − sample/control] × 100.

### 2.6. Reactive Oxygen Species Measurement

The intracellular level of ROS was determined by the indicator 6-carboxy-2′,7′-dichlorodihydrofluorescein diacetate (DCFDA), while mitochondria oxidative stress was evaluated by MitoSOX (Thermo fisher). After the treatment, the medium was removed, and the cells were incubated with 20 µM carboxy-DCFDA and 500 nM MitoSOX at 37 °C in the dark for 30 min.

The fluorescence intensity was measured using Thermo Varioskan Flash (Thermo Electron Corporation, Vantaa, Finland) with excitation/emission settings of 492/515 nm (ROS) and 392/610 nm (MitoSOX), respectively.

### 2.7. Experimental Animal Model

Forty male Sprague Dawley (SD) rats (5 weeks old) were acclimatized for one week before the experimental study. All animal care and experimental procedures strictly adhered to guidelines and regulations approved by the Institutional Animal Care and Use Committee at Taipei Medical University (LAC-2022-0148). The rats were exposed to 12 h light/12 h dark natural day and night cycles and allowed ad libitum access to a standard chow diet and water. They were randomly divided into 4 groups of 8–10 rats each, comprising the control group (CON): treated with vehicle; cisplatin group (CIS): treated with CIS (10 mg/kg bw, i.p.); low-L-cysteine group (LC): treated with CYS 100 mg/kg bw/day by gavage + CIS (10 mg/kg bw, i.p.); and high-L-cysteine group (HC): treated with CYS 300 mg/kg bw/day by gavage + CIS (10 mg/kg bw, i.p.) [19]. CYS administrations were conducted for 21 consecutive days; CIS (10 mg/kg body weight, intraperitoneal) was injected to establish a reproductive damage model at day 17 [20]. After the treatment, the SD rats were weighed and anesthetized. A combination of Zoletil (20 mg/kg) and xylazine (5 mg/kg) was administered to induce anesthesia, ensuring unconsciousness while maintaining cardiac activity. Serum, testis, and sperm collection was performed for analysis.

### 2.8. Hematoxylin and Eosin (H&E) Staining

The testes were fixed in 10% buffered formalin solution, dehydrated, and then embedded in paraffin. The embedding and Hematoxylin and Eosin (H&E) staining processes were outsourced to BIO-CHECK LABORATORIES LTD (New Taipei City, Taiwan). The testis sections were then assessed using H&E staining for histopathological investigations.

### 2.9. Sperm Analysis

The epididymis was tested to determine the sperm parameters. Initially, the epididymis was cut twice in 4 mL DMEM/F12 (phenol red free) and then incubated at 37 °C for 5 min. A 10 μL aliquot of the sperm suspension was introduced into a Neubauer chamber and examined under a 100× light microscope to assess sperm count. This chamber was also utilized to evaluate sperm viability, expressed as viability percentages (%), where non-motile, immotile sperms were classified as dead. For the analysis of sperm morphological abnormality, the 5–20 μL sperm suspension was placed on the slides and smeared. After the slides dried naturally, they were stained with the modified Diff-Quik (Eosin Y and crystal violet) method [21,22] and examined under a 400× light microscope to assess morphological abnormalities [23].

### 2.10. Hormonal and Biochemical Estimations

Blood serum was collected from the cardiac puncture after the treatment and then centrifuged (3500× *g*, 4 °C) for 10 min to obtain serum. The serum levels of testosterone, serum glutamic-oxalocetic transaminase (SGOT), serum glutamic-pyruvic transaminase (SGPT), blood urea nitrogen (BUN), creatinine, uric acid (UA), lactate dehydrogenase (LDH), and creatine kinase (CPK) were delivered on the day to Le Zen Reference Lab (Taipei, Taiwan) for hormonal and biochemical examinations.

### 2.11. Western Blot Analysis

The cells and testis tissues were lysed in lysis buffer containing radioimmunoprecipitation assay (RIPA) buffer and protease and phosphatase inhibitors. Sample protein quantification in the supernatant was determined using a T-Pro bicinchoninic acid (BCA) Assay Kit. The cell (30 µg) and tissue (60 µg) proteins were separated by sodium dodecyl sulfate polyacrylamide gel electrophoresis (SDS-PAGE) and electrotransferred to a polyvinylidene fluoride (PVDF) membrane. After the membranes were blocked for 1 h with blocking buffer (5% BSA) at room temperature, the membranes were incubated with primary antibodies at 4 °C overnight, including Glyceraldehyde 3-phosphate dehydrogenase (GAPDH) (HRP-60004, Proteintech (Rosemont, IL, USA)), HO-1 (10701-1-AP, Proteintech), Bcl-2 associated X (Bax) (2772, Cell Signaling (Danvers, MA, USA)), B-cell lymphoma-2 (Bcl-2) (68103-1-lg, Proteintech), Caspase 3 (9662, Cell Signaling), poly ADP-ribose polymerase (PARP) (9542, Cell signaling), Inducible nitric oxide synthase (iNOS) (18985-1-AP, Proteintech), Cyclooxygenase 2 (COX2) (160106, Cayman), Zonula occludens-1 (ZO-1) (AF5145, Affinity (Cincinnati, OH, USA)), Occludin (DF7504, Affinity), and P450scc (13363-1-AP, Proteintech). Subsequently, the membranes were incubated with a horseradish peroxidase (HRP)-conjugated secondary antibody (1:5000–10,000) at room temperature for 2 h and then washed three times with Tris-buffered saline with Tween 20 (TBST). The bands were visualized using enhanced chemiluminescence (ECL) reagent and captured by an image analysis system [24] (eBLOT Touch Imager (eBlot Photoelectric Technology, Shanghai, China)). The densitometric values were normalized to the internal control GAPDH and quantified by ImageJ software (Version 1.54f).

### 2.12. Statistical Analysis

All results are expressed as mean ± standard deviation (SD) for in vitro data or standard error of the mean (SEM) for animal data. Data were normalized where appropriate and analyzed using GraphPad Prism 9.0. Differences among multiple groups were analyzed using one-way ANOVA followed by Tukey’s multiple comparison test, while differences between two groups were tested using a two-tailed Student’s *t*-test. The Shapiro–Wilk test was used to confirm normality [25]. A *p* value < 0.05 was considered statistically significant.

## 3. Results

### 3.1. Effect of L-Cysteine and Cisplatin on TM3 and TM4 Cell Viability

**Figure 1:**
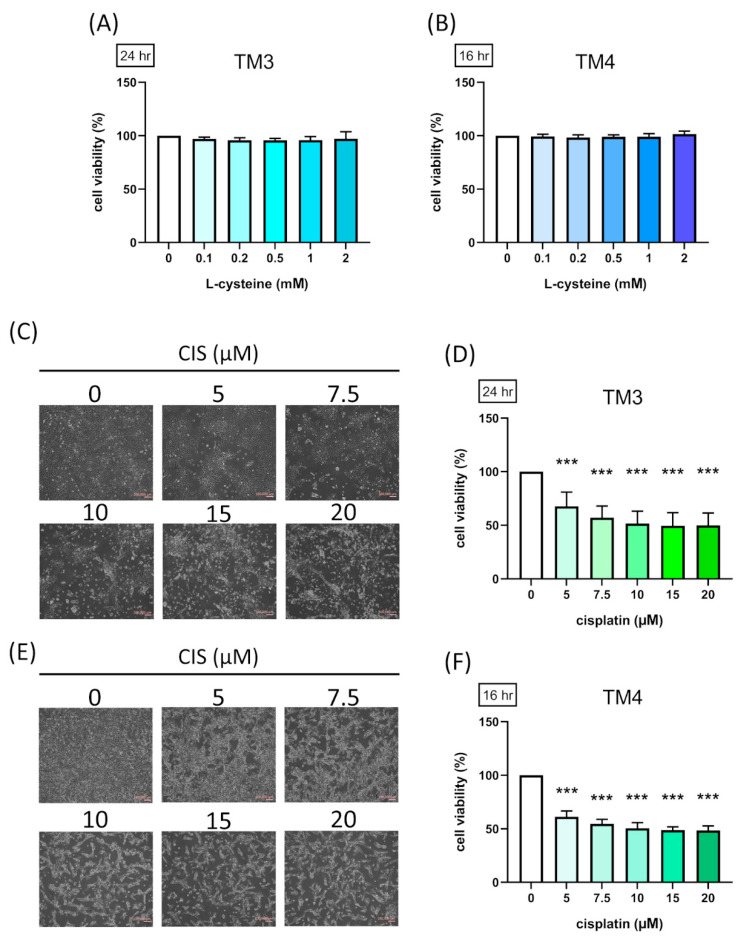
Effect of L-cysteine (CYS) and cisplatin (CIS) on TM3 and TM4 cell viability. TM3 (5000 cells/well) (n = 7) and TM4 cells (3000 cells/well) (n = 7) were cultured in a 96-well plate for cell viability assays. Cells were treated with different concentrations of (**A**,**B**) CYS (0.1, 0.2, 0.5, 1, and 2 mM) or (**D**,**F**) CIS (5, 7.5, 10, 15, and 20 µM) for 24 or 16 h and stained with MTS assay. (**C**) TM3 and (**E**) TM4 cells (4 × 10^4^ cell/well) were cultured in a 12-well plate for cell morphology assessment. Scale bar: 100 μm. Data are represented as mean ± SD. Different groups were analyzed using one-way ANOVA following by Tukey’s multiple comparison test. *** *p* < 0.001 compared with the control group.

To explore the modulatory role of CYS in Leydig and Sertoli cells, we conducted experiments using TM3 and TM4 cell models. Cell viability was evaluated using the MTS Assay Kit. TM3 and TM4 cells were treated with different concentrations of CYS (0.1, 0.2, 0.5, 1, and 2 mM) for 24 h (0.1 mM: 97.00 ± 1.51 (*p* value = 0.36 compared with control group); 0.2 mM: 95.71 ± 2.05 (*p* value = 0.07 compared with control group); 0.5 mM: 95.57 ± 1.76 (*p* value = 0.056 compared with control group); 1 mM: 95.83 ± 3.02 (*p* value = 0.11 compared with control group); 2 mM: 97.14 ± 6.03 (*p* value = 0.42 compared with control group)) or 16 h (0.1 mM: 99.21 ± 2.04 (*p* value = 0.94 compared with control group); 0.2 mM: 98.42 ± 2.29 (*p* value = 0.44 compared with control group); 0.5 mM: 98.86 ± 1.81 (*p* value = 0.76 compared with control group); 1 mM: 99.00 ± 2.83 (*p* value = 0.85 compared with control group); 2 mM: 101.5 ± 2.75 (*p* value = 0.50 compared with control group)). The results showed that CYS did not affect the cell viability of TM3 and TM4 cells (Figure 1A,B), indicating that CYS is generally safe for both reproductive cell types. To establish the damage condition of cisplatin on TM3 and TM4 cells, we observed that cell morphology significantly shrank compared to the control group (Figure 1C,E). TM3 and TM4 cells were treated with different concentrations of CIS (5, 7.5, 10, 15, and 20 µM) for 24 h (5 µM: 67.64 ± 13.25; 7.5 µM: 56.91 ± 11.04; 10 µM: 51.55 ± 11.58; 15 µM: 49.45 ± 12.15; 20 µM: 49.73 ± 11.74. *p* value less than 0.001) or 16 h (5 µM: 62.00 ± 6.02; 7.5 µM: 55.36 ± 4.15; 10 µM: 50.64 ± 5.78; 15 µM: 49.00 ± 3.41; 20 µM: 48.09 ± 4.68. *p* value less than 0.001). The results showed a significant reduction in cell viability, with 50% viability loss observed in TM3 cells and in TM4 cells, demonstrating a dose-dependent effect (Figure 1D,F). These findings suggest that the effective concentrations of CYS and CIS induced harmfulness.

### 3.2. L-Cysteine Attenuates Cisplatin-Induced Reduction in TM3 and TM4 Cell Viability

**Figure 2:**
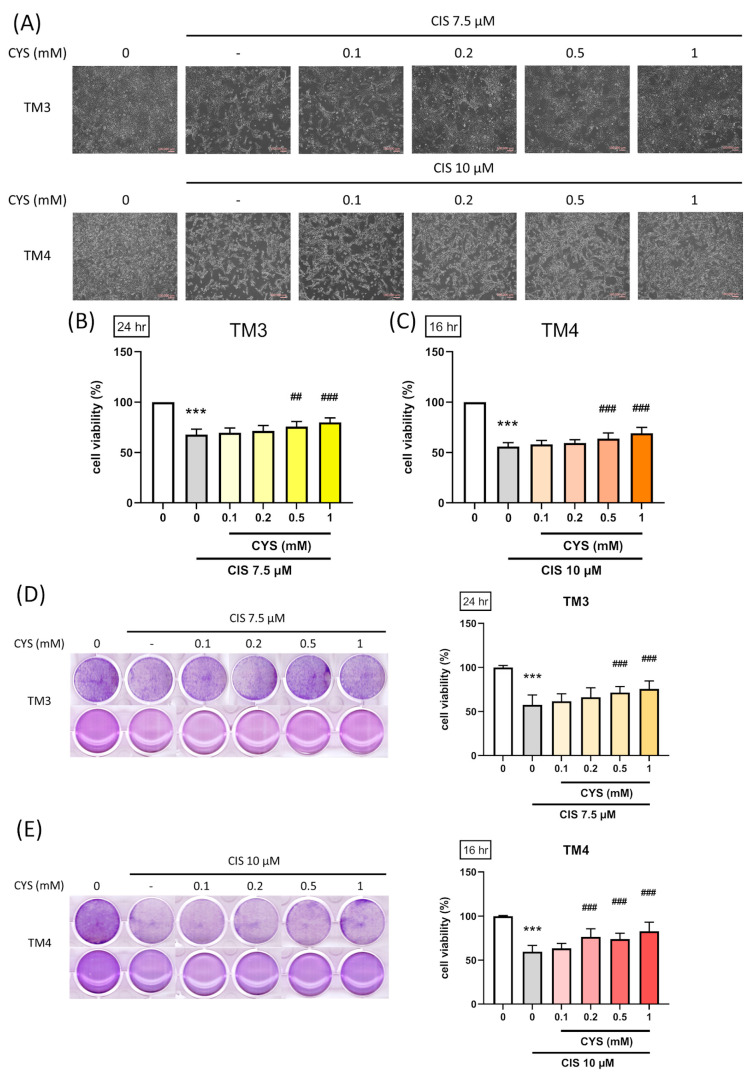
L-cysteine (CYS) attenuates cisplatin (CIS)-induced reduction in TM3 and TM4 cell viability. TM3 (5000 cell/well) (n = 7) and TM4 cells (3000 cell/well) (n = 7) were cultured in a 96-well plate for cell viability. TM3 (n = 7) and TM4 (n = 7) cells (4 × 10^4^ cell/well) were cultured in a 12-well plate for cell morphology and composition. They were co-treated with different concentrations of CYS (0.1, 0.2, 0.5, 1 mM) and, respectively, induced by CIS (7.5 or 10 µM) for 24 and 16 h. (**A**) Morphology, (**B**,**C**) viability, and (**D**,**E**) crystal violet staining in TM3 and TM4 cells. Scale bar: 100 μm. Data are represented as mean ± SD. Different groups were analyzed using one-way ANOVA following by Tukey’s multiple comparison test. *** *p* < 0.001 compared with the control group. ## *p* < 0.01 and ### *p* < 0.001 compared with CIS group.

To understand the effect of CYS on cell viability and morphology in cisplatin-induced cell damage. We used the MTS assay kit and crystal violet staining to evaluate cell viability and composition. TM3 and TM4 cells were co-treated with different concentrations of CYS (0.1, 0.2, 0.5, and1 mM) and induced with CIS (7.5 or 10 µM) for 24 h (CIS group: 67.80 ± 5.29 (*p* value < 0.0001 compared with the control group); 0.1 mM: 69.70 ± 4.69 (*p* value = 0.93 compared with CIS group); 0.2 mM: 71.50 ± 5.28 (*p* value = 0.49 compared with CIS group); 0.5 mM: 75.70 ± 4.99 (*p* value = 0.003 compared with CIS group); 1 mM: 80.00 ± 4.29 (*p* value < 0.0001 compared with CIS group)) and 16 h (CIS group: 55.93 ± 3.89 (*p* value < 0.0001 compared with the control group); 0.1 mM: 59.50 ± 3.18 (*p* value = 0.95 compared with CIS group); 0.2 mM: 59.50 ± 3.18 (*p* value = 0.95 compared with CIS group); 0.5 mM: 63.86 ± 5.56 (*p* value = 0.35 compared with CIS group); 1 mM: 69.14 ± 5.75 (*p* value = 0.0093 compared with CIS group)), respectively. The results showed that 0.5 and 1 mM CYS significantly restored cell morphology (Figure 2A) and cell viability (Figure 2B,C). Crystal violet staining revealed consistent results in both cell types (Figure 2D,E) (TM3: CIS group: 57.64 ± 11.19 (*p* value < 0.0001 compared with the control group); 0.1 mM: 61.71 ± 8.34 (*p* value = 0.81 compared with CIS group); 0.2 mM: 66.28 ± 10.67 (*p* value = 0.09 compared with CIS group); 0.5 mM: 71.57 ± 6.79 (*p* value = 0.0007 compared with CIS group); 1 mM: 75.71 ± 8.99 (*p* value < 0.0001 compared with CIS group)) (TM4: CIS group: 59.58 ± 7.17 (*p* value < 0.0001 compared with the control group); 0.1 mM: 63.42 ± 5.60 (*p* value = 0.79 compared with CIS group); 0.2 mM: 76.58 ± 9.04 (*p* value < 0.0001 compared with CIS group); 0.5 mM: 74.00 ± 6.63 (*p* value < 0.0001 compared with CIS group); 1 mM: 82.92 ± 10.26 (*p* value < 0.0001 compared with CIS group). These findings indicate the protective potential of CYS on CIS-induced cell damage.

### 3.3. Antioxidative Ability of L-Cysteine Suppresses Reactive Oxygen Species Production in Cisplatin-Induced TM3 and TM4 Cells

**Figure 3:**
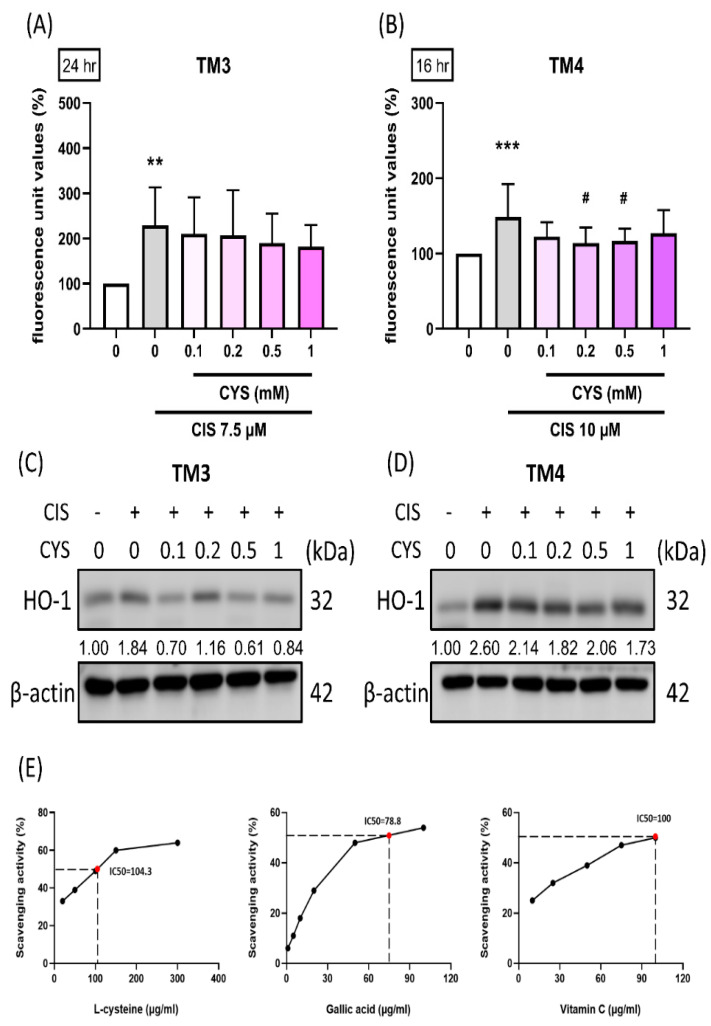
Effect of L-cysteine (CYS) on reactive oxygen species production in cisplatin (CIS)-induced TM3 and TM4 cells. The cells (1.2 × 10^5^ cells/well) were cultured in a 6-well plate. They were co-treated with different concentrations of CYS (0.1, 0.2, 0.5, and 1 mM) and, respectively, induced by CIS (7.5 or 10 µM) for 24 and 16 h (n = 7). (**A**,**B**) ROS measurements were conducted by incubating the cells with 20 μM of DCFDA for 30 min at 37 °C in the dark. (**C**,**D**) HO-1 expression was analyzed by Western blot. (**E**) DPPH radical scavenging activity (%) of L-cysteine, gallic acid, and vitamin C. The IC50 DPPH values were obtained through extrapolation from regression analysis. Data are represented as mean ± SD. Different groups were analyzed using one-way ANOVA followed by Tukey’s multiple comparison test. ** *p* < 0.01 and *** *p* < 0.001 compared with the control group. # *p* < 0.05 compared with the CIS group.

To explore the mechanism leading to cell damage, we examined the relationship between cisplatin-induced cytotoxicity and reactive oxygen species (ROS) generation using the indicator 2′,7′-dichlorodihydrofluorescein diacetate (DCFDA). The results showed that CIS treatment significantly increased ROS fluorescence unit values. Moreover, CYS partially reduced ROS production in both cisplatin-induced cell types (Figure 3A,B) (TM3: CIS group: 228.56 ± 84.01 (*p* value = 0.0047 compared with the control group); 0.1 mM: 210.00 ± 81.18 (*p* value = 0.99 compared with CIS group); 0.2 mM: 206.44 ± 100.72 (*p* value = 0.99 compared with CIS group); 0.5 mM: 189.33 ± 65.34 (*p* value = 0.85 compared with CIS group); 1 mM: 181.67 ± 47.90 (*p* value = 0.73 compared with CIS group))(TM4: CIS group: 148.55 ± 43.75 (*p* value < 0.0001 compared with the control group); 0.1 mM: 122.55 ± 18.92 (*p* value = 0.18 compared with CIS group); 0.2 mM: 113.64 ± 20.76 (*p* value = 0.03 compared with CIS group); 0.5 mM: 116.36 ± 16.50 (*p* value = 0.049 compared with CIS group); 1 mM: 126.91 ± 30.66 (*p* value = 0.36 compared with CIS group) and decreased the expression of activated HO-1 (Figure 3C,D). We used the DPPH assay to examine the antioxidant activity of CYS. Our data revealed that the DPPH antioxidant activity of CYS had an IC50 value of 104.3 μg/mL, demonstrating its antioxidative capacity, especially when compared with the reference antioxidants gallic acid and vitamin C (Figure 3E).

### 3.4. L-Cysteine Attenuates Apoptosis in Cisplatin-Induced TM3 and TM4 Cells

**Figure 4:**
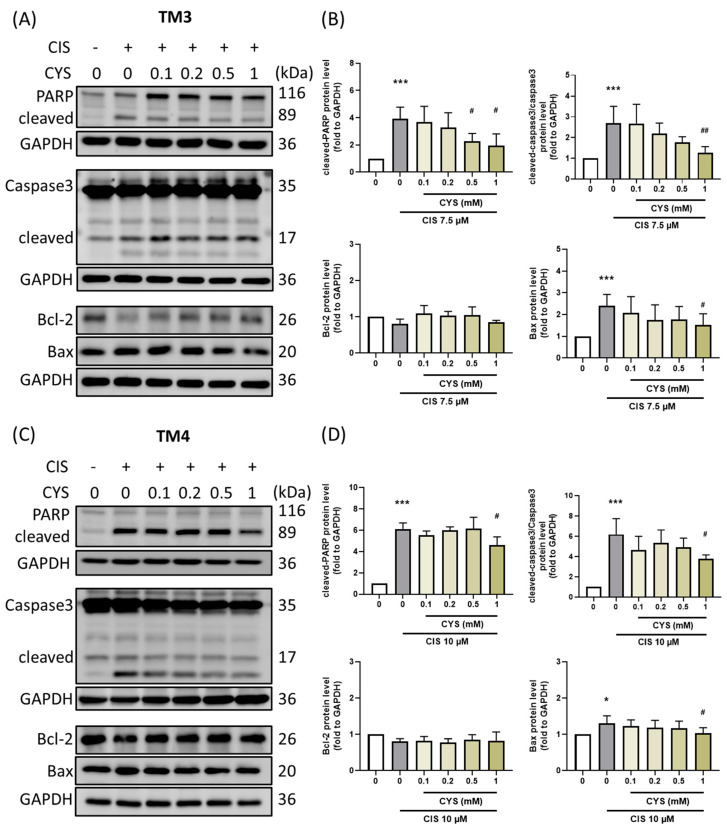
Effect of L-cysteine (CYS) on apoptosis-related protein expression in cisplatin (CIS)-induced TM3 and TM4 cells. (**A**) TM3 and (**C**) TM4 cells (1 × 10^5^ cells) were cultured in a 6 cm dish. Cells were co-treated with different concentrations of CYS (0.1, 0.2, 0.5, and 1 mM) and induced with CIS (7.5 or 10 µM) for 24 and 16 h, respectively (n = 4). The protein expression of PARP, Caspase 3, Bcl-2, and Bax was determined by Western blot, and the band values were normalized to GAPDH. (**B**,**D**) Quantification of protein expression. Data are represented as mean ± SD. Different groups were analyzed using one-way ANOVA followed by Tukey’s multiple comparison test. * *p* < 0.05 and *** *p* < 0.001 compared with the control group. # *p* < 0.05 and ## *p* < 0.01 compared with the CIS group.

Oxidative stress-induced apoptosis occurs in CIS-induced testicular damage [26]. Treatment with 1 mM CYS significantly alleviated CIS-induced expression of PARP (TM3: CIS group: 3.92 ± 0.85 (*p* value = 0.0002 compared with the control group); 0.1 mM: 3.69 ± 1.14 (*p* value = 0.99 compared with CIS group); 0.2 mM: 3.26 ± 1.10 (*p* value = 0.82 compared with CIS group); 0.5 mM: 2.26 ± 0.58 (*p* value = 0.049 compared with CIS group); 1 mM: 1.95 ± 0.86 (*p* value = 0.013 compared with CIS group)) (TM4: CIS group: 6.12 ± 0.57 (*p* value < 0.0001 compared with the control group); 0.1 mM: 5.54 ± 0.39 (*p* value = 0.77 compared with CIS group); 0.2 mM: 6.00 ± 0.31 (*p* value = 0.99 compared with CIS group); 0.5 mM: 6.15 ± 1.07 (*p* value = 0.99 compared with CIS group); 1 mM: 4.60 ± 0.78 (*p* value = 0.03 compared with CIS group)), Caspase 3 (TM3: CIS group: 2.69 ± 0.82 (*p* value = 0.0002 compared with the control group); 0.1 mM: 2.68 ± 0.92 (*p* value = 0.99 compared with CIS group); 0.2 mM: 2.20 ± 0.50 (*p* value = 0.70 compared with CIS group); 0.5 mM: 1.78 ± 0.26 (*p* value = 0.11 compared with CIS group); 1 mM: 1.28 ± 0.29 (*p* value = 0.002 compared with CIS group)) (TM4: CIS group: 6.19 ± 1.55 (*p* value < 0.0001 compared with the control group); 0.1 mM: 4.62 ± 1.37 (*p* value = 0.26 compared with CIS group); 0.2 mM: 5.35 ± 1.27 (*p* value = 0.83 compared with CIS group); 0.5 mM: 4.93 ± 0.88 (*p* value = 0.49 compared with CIS group); 1 mM: 3.79 ± 0.38 (*p* value = 0.017 compared with CIS group)), Bcl-2 (TM3: CIS group: 0.81 ± 0.12 (*p* value = 0.49 compared with the control group); 0.1 mM: 1.09 ± 0.22 (*p* value = 0.14 compared with CIS group); 0.2 mM: 1.03 ± 0.12 (*p* value = 0.35 compared with CIS group); 0.5 mM: 1.05 ± 0.22 (*p* value = 0.24 compared with CIS group); 1 mM: 0.85 ± 0.05 (*p* value = 0.99 compared with CIS group)) (TM4: CIS group: 0.80 ± 0.08 (*p* value = 0.23 compared with the control group); 0.1 mM: 0.82 ± 0.13 (*p* value = 0.99 compared with CIS group); 0.2 mM: 0.77 ± 0.11 (*p* value = 0.99 compared with CIS group); 0.5 mM: 0.85 ± 0.14 (*p* value = 0.99 compared with CIS group); 1 mM: 0.83 ± 0.24 (*p* value = 0.99 compared with CIS group)), and Bax (TM3: CIS group: 2.39 ± 0.48 (*p* value = 0.001 compared with the control group); 0.1 mM: 1.00 ± 0.00 (*p* value = 0.94 compared with CIS group); 0.2 mM: 1.00 ± 0.00 (*p* value = 0.52 compared with CIS group); 0.5 mM: 1.78 ± 0.53 (*p* value = 0.51 compared with CIS group); 1 mM: 1.00 ± 0.00 (*p* value = 0.16 compared with CIS group)) (TM4: CIS group: 1.30 ± 0.21 (*p* value = 0.012 compared with the control group); 0.1 mM: 1.23 ± 0.17 (*p* value = 0.95 compared with CIS group); 0.2 mM: 1.19 ± 0.20 (*p* value = 0.75 compared with CIS group); 0.5 mM: 1.17 ± 0.19 (*p* value = 0.63 compared with CIS group); 1 mM: 1.03 ± 0.15 (*p* value = 0.03 compared with CIS group)) proteins (Figure 4). These results suggest that CYS may reverse cisplatin-induced cell apoptosis and thereby restore testicular function.

### 3.5. L-Cysteine Reduces Inflammatory Responses in Cisplatin-Induced TM3 Cells

**Figure 5:**
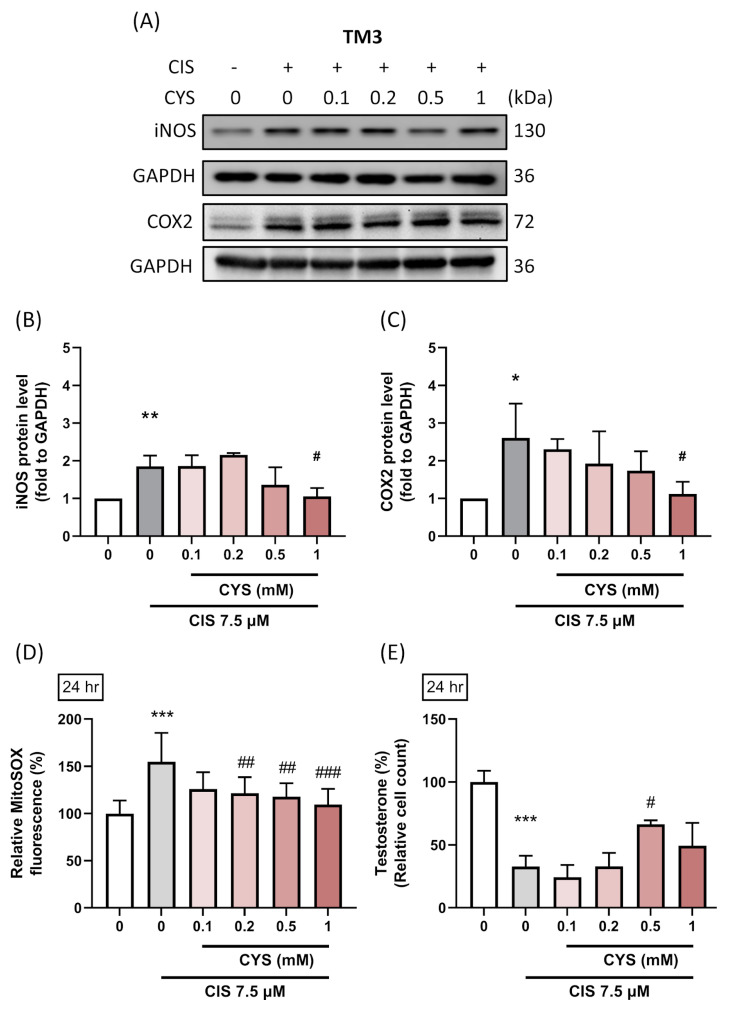
Effect of L-cysteine (CYS) on testosterone secretion-related modulators in cisplatin (CIS)-induced TM3 cells. TM3 cells (1 × 10^5^ cell/well) were cultured in a 6 cm dish. Cells were co-treated with different concentrations of CYS (0.1, 0.2, 0.5, 1 mM) and induced with CIS (7.5 µM) for 24 h (n = 4). (**A**) The inflammatory protein expressions were determined by Western blot. (**B**) iNOS and (**C**) COX2 protein expression levels were normalized to GAPDH. (**D**) Mitochondrial oxidative stress was evaluated by MitoSOX staining, with fluorescence intensity. (**E**) Testosterone secretion in the culture medium was quantified using an ELISA kit. Data are represented as mean ± SD. Different groups were analyzed using one-way ANOVA followed by Tukey’s multiple comparison test. * *p* < 0.05, ** *p* < 0.01 and *** *p* < 0.001 compared with the control group. # *p* < 0.05, ## *p* < 0.01 and ### *p* < 0.001 compared with the CIS group.

Given the significant cell damage and apoptosis observed, we further evaluated the expression of inflammation-related proteins. The results showed that TM3 cells were more sensitive to inflammation in the cisplatin group, which significantly activated iNOS (CIS group: 1.85 ± 0.28 (*p* value = 0.01 compared with the control group); 0.1 mM: 1.86 ± 0.29 (*p* value = 0.99 compared with CIS group); 0.2 mM: 2.16 ± 0.05 (*p* value = 0.73 compared with CIS group); 0.5 mM: 1.37 ± 0.46 (*p* value = 0.29 compared with CIS group); 1 mM: 1.06 ± 0.22 (*p* value = 0.03 compared with CIS group)) and COX2 (CIS group: 2.61 ± 0.91 (*p* value = 0.014 compared with the control group); 0.1 mM: 2.30 ± 0.27 (*p* value = 0.98 compared with CIS group); 0.2 mM: 1.92 ± 0.86 (*p* value = 0.65 compared with CIS group); 0.5 mM: 1.74 ± 0.51 (*p* value = 0.33 compared with CIS group); 1 mM: 1.12 ± 0.32 (*p* value = 0.04 compared with CIS group)) protein expression compared to the control group. Additionally, 1 mM CYS significantly decreased the expression of these inflammation-related proteins (Figure 5). This indicates that CYS can significantly reduce the risk of inflammation in CIS-induced TM3 cells. Accompanied by the recovery of mitochondrial oxidative stress (MitoSOX) (CIS group: 154.91 ± 30.50 (*p* value < 0.0001 compared with the control group); 0.1 mM: 125.91 ± 17.84 (*p* value = 0.009 compared with CIS group); 0.2 mM: 121.455 ± 16.90 (*p* value = 0.0017 compared with CIS group); 0.5 mM: 117.73 ± 14.36 (*p* value = 0.004 compared with CIS group); 1 mM: 109.55 ± 16.47 (*p* value < 0.0001 compared with CIS group))and testosterone secretion (CIS group: 32.85 ± 8.51 (*p* value < 0.0001 compared with the control group); 0.1 mM: 24.17 ± 9.53 (*p* value = 0.92 compared with CIS group); 0.2 mM: 33.25 ± 10.53 (*p* value = 0.99 compared with CIS group); 0.5 mM: 66.55 ± 3.25 (*p* value = 0.023 compared with CIS group); 1 mM: 48.98 ± 18.04 (*p* value = 0.45 compared with CIS group)), these results indicate the protective role of CYS in the improvement in reproductive function.

### 3.6. L-Cysteine Restored Blood–Testis Barrier Functional Protein Expression in Cisplatin-Induced TM4 Cells

**Figure 6:**
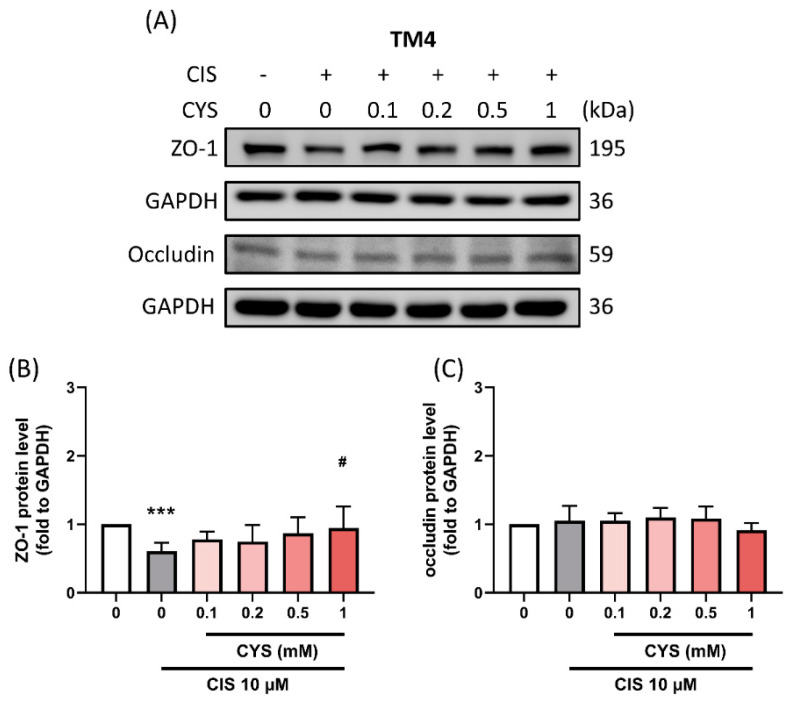
Effect of L-cysteine (CYS) on blood–testis barrier (BTB)-related protein expression in cisplatin (CIS)-induced TM4 cells. The cells (1 × 10^5^ cell/well) were cultured in a 6 cm dish. Cells were co-treated with different concentrations of CYS (0.1, 0.2, 0.5, and 1 mM) and induced with CIS (10 µM) for 24 h (n = 4). (**A**) The BTB protein expressions were determined by Western blot. (**B**) ZO-1 and (**C**) occludin protein expression levels were normalized to GAPDH. Data are represented as mean ± SD. Different groups were analyzed using one-way ANOVA followed by Tukey’s multiple comparison test. *** *p* < 0.001 compared with the control group. # *p* < 0.05 compared with the CIS group.

The blood–testis barrier (BTB) can confer protective effects that decrease cisplatin toxicity to the testicles [27]. To examine the integrity of the BTB structure, we evaluated the expression of tight-junction-related proteins by Western blotting. The results showed that CYS treatment significantly recovered ZO-1 protein expression (CIS group: 0.61 ± 0.13 (*p* value = 0.001 compared with the control group); 0.1 mM: 0.78 ± 0.12 (*p* value = 0.58 compared with CIS group); 0.2 mM: 0.75 ± 0.24 (*p* value = 0.75 compared with CIS group); 0.5 mM: 0.87 ± 0.23 (*p* value = 0.11 compared with CIS group); 1 mM: 0.95 ± 0.31 (*p* value = 0.017 compared with CIS group)) (Figure 6). However, neither CIS nor CYS affected Occludin protein expression (CIS group: 1.05 ± 0.22 (*p* value = 0.99 compared with the control group); 0.1 mM: 1.05 ± 0.12 (*p* value = 0.99 compared with CIS group); 0.2 mM: 1.10 ± 0.14 (*p* value = 0.99 compared with CIS group); 0.5 mM: 1.08 ± 0.18 (*p* value = 0.99 compared with CIS group); 1 mM: 1.07 ± 0.87 (*p* value = 0.50 compared with CIS group)). These results suggest that CYS only partially reverses the impairment in BTB structure (ZO-1) in CIS-induced TM4 cells.

### 3.7. Effect of L-Cysteine on Cisplatin-Induced Testicular Toxicity in In Vivo Study

**Figure 7:**
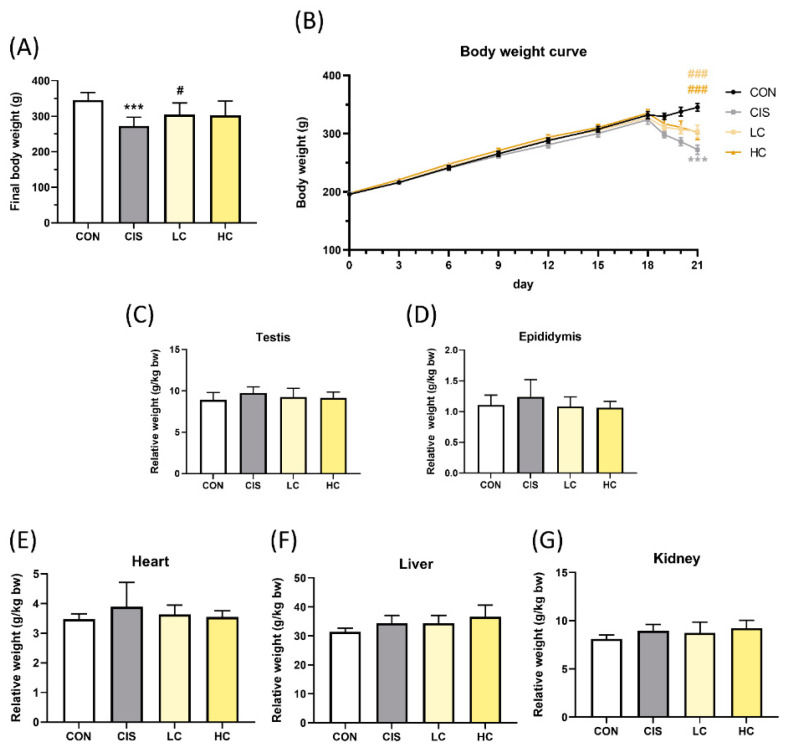
Effect of L-cysteine (CYS) on cisplatin (CIS)-induced weight and organ changes in in vivo model. (**A**) Body weight and (**B**) body weight changes, and the relative weight changes of the (**C**) testis, (**D**) epididymis, (**E**) heart, (**F**) liver, and (**G**) kidney in a CIS-induced animal model after CYS pretreatment (n = 8). Data are represented as mean ± SEM. Different groups were analyzed using Student’s *t*-test. *** *p* < 0.001 compared with the CON group. # *p* < 0.05 and ### *p* < 0.001 compared with the CIS group. CON: control; CIS: cisplatin (10 mg/kg bw); LC: low CYS (100 mg/kg bw); HC: high CYS (300 mg/kg bw).

To investigate the effect of CYS on CIS-induced testicular toxicity in an animal model, we used a single dose of 10 mg/kg of CIS injected into rats to define the testicular toxicity model [28,29,30]. The SD rats were administered low CYS (LC, 100 mg/kg bw/day) or high CYS (HC, 300 mg/kg bw/day) for 21 consecutive days and then injected with CIS (10 mg/kg bw, single intraperitoneal injection) on the 17th day. CYS treatment significantly alleviated body weight loss, which caused a significant reduction within the cisplatin group (Figure 7A,B) (CIS group: 272.35 ± 7.86 (*p* value < 0.0001 compared with CON group (345.35 ± 6.78); LC group: 304.00 ± 10.63 (*p* value = 0.014 compared with CIS group); HC group: 302.35 ± 12.86 (*p* value = 0.011 compared with CIS group)). This treatment did not cause significant organ weight changes after adjusting for body weight (Figure 7C–G). Additionally, compared to the CON group, the CIS, LC, and HC group rats exhibited significant weakness, poor appetite, indigestion, and diarrhea.

### 3.8. Effects of L-Cysteine on Testis, Liver, Kidney, and Heart Functional Biochemical Indicators in Cisplatin-Induced Male Rats

**Figure 8:**
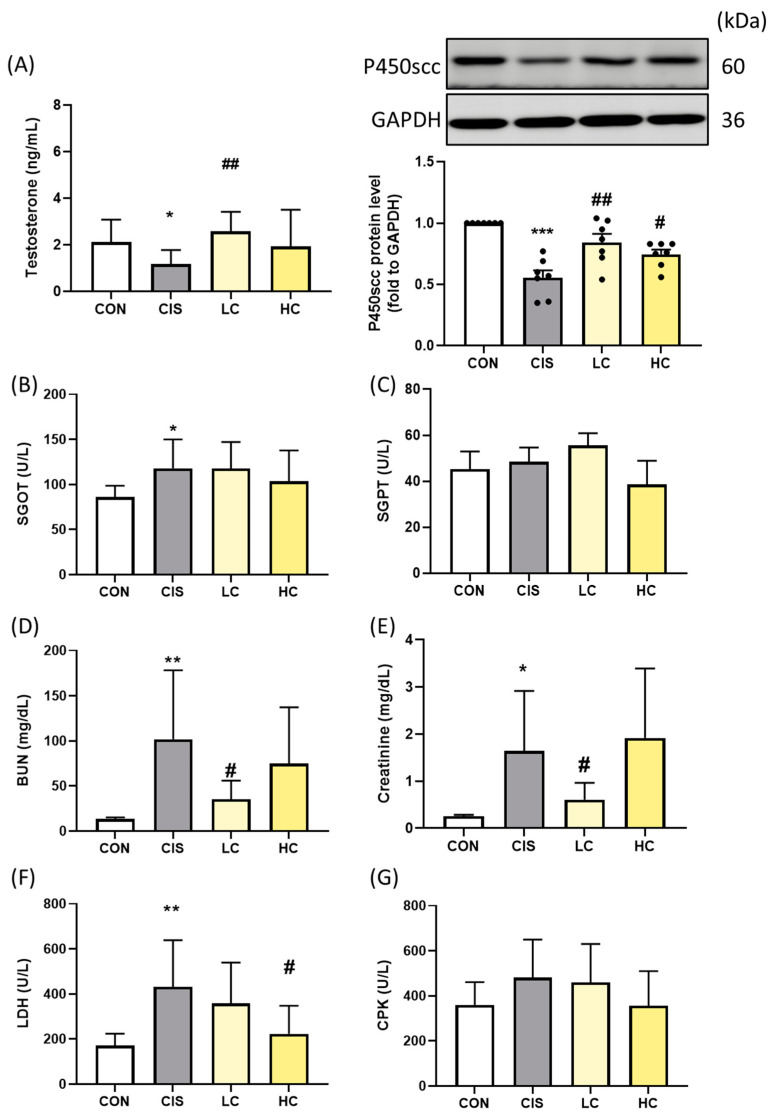
Protective role of L-cysteine (CYS) treatment for the biochemical indicators in cisplatin-induced in vivo model. (**A**) Testosterone and P450scc protein expression were evaluated by Western blot; (**B**) SGOT, (**C**) SGPT, (**D**) BUN, (**E**) creatinine, (**F**) LDH, and (**G**) CPK levels in a cisplatin-induced animal model after CYS pretreatment (n = 8). Data are represented as mean ± SEM. Different groups were analyzed using Student’s *t*-test. * *p* < 0.05, ** *p* < 0.01, and *** *p* < 0.001 compared with the CON group. # *p* < 0.05 and ## *p* < 0.01 compared with the CIS group. CON: control; CIS: cisplatin (10 mg/kg bw); LC: low CYS (100 mg/kg bw); and HC: high CYS (300 mg/kg bw).

To evaluate the systemic effects of cisplatin toxicity and the protective role of L-cysteine (CYS) treatment, we assessed serum biomarkers of organ function. Testosterone levels were significantly elevated in the LC group compared to the CIS group (Figure 8A). Specifically, the CIS group exhibited a testosterone level of 1.17 ± 0.23, which was significantly lower than the CON group (2.12 ± 0.36, *p* = 0.05). In contrast, the LC group showed a marked recovery with a testosterone level of 2.58 ± 0.32 (*p* = 0.004 compared with the CIS group), while the HC group had a testosterone level of 1.93 ± 0.60 (*p* = 0.26 compared with CIS). This recovery in testosterone production was accompanied by the restoration of P450scc (CYP11A1) protein expression, indicating the restoration of steroidogenesis. In addition, CIS treatment caused damage to liver, kidney, and heart functions, as evidenced by elevated serum markers. Liver function markers (SGOT), kidney function markers (BUN and creatinine), and heart function markers (LDH and CPK) were significantly impaired in the CIS group compared to the CON group. Notably, CYS treatment led to a partial recovery of these functions (Figure 8B–G). These findings highlight the protective potential of CYS in mitigating organ dysfunction induced by cisplatin toxicity.

### 3.9. L-Cysteine Recovers Testicular Histopathology in Cisplatin-Induced Male Rats

**Figure 9:**
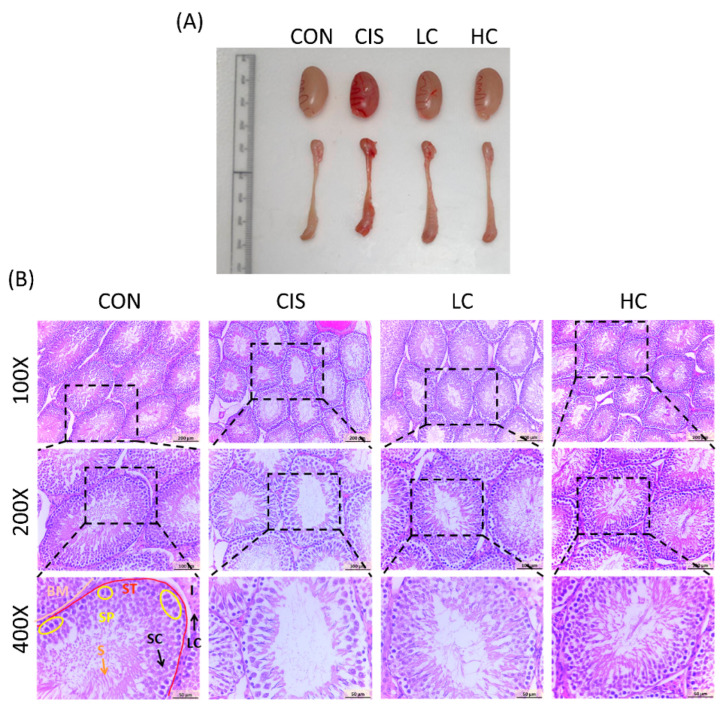
Effect of L-cysteine (CYS) on testicular damage in cisplatin (CIS)-induced male rats. (**A**) The appearance of the testis and epididymis. (**B**) Hematoxylin-and-eosin-stained sections of the testis, observed under magnification at ×100, ×200, and ×400. Observations were made on multiple sections (2–3) from each testicular fragment to ensure consistent and representative assessment. Labels in the figure include Basement Membrane (BM, beige line), seminiferous tubules (STs, red line), Sertoli cells (SCs), sperm (S), spermatocytes (SPs, yellow circle), Interstitial Tissue (I), and Leydig cells (LCs). Treatment groups: CON (control), CIS (cisplatin, 10 mg/kg bw), LC (low CYS, 100 mg/kg bw), and HC (high CYS, 300 mg/kg bw).

To investigate the cause of the reduced testosterone levels following cisplatin (CIS) treatment, we hypothesized that structural damage to the testis could impact hormone secretion. Macroscopic observations of the testes and epididymis were taken (Figure 9A). The testes and epididymis in the CIS group appeared redder compared to the control and treatment groups, suggesting potential vascular alterations or inflammation induced by cisplatin [31]. Testis tissue was observed through H&E staining sections. The results showed that the control testis tissue had normal seminiferous tubules and an orderly arrangement of germinal cells. In the germinal layer, spermatogonia, Sertoli cells, primary spermatocytes, and spermatids were aligned in a structured pattern toward the lumen. Leydig cells in the interstitial space showed mild denaturation and atrophy compared to controls [32]. These observations align with cisplatin’s known testicular toxicity and potential steroidogenic disruption. Conversely, treatment with CYS markedly mitigated these histological changes, restoring near-normal architecture of the seminiferous tubules, with well-arranged Sertoli and Leydig cells, as well as visible spermatogenic cells within the lumen (Figure 9B). These results suggest that the histological structure of CIS-induced testes improves with CYS treatment.

### 3.10. L-Cysteine Ameliorates Sperm Morphology and Viability in Cisplatin-Induced Male Rats

**Figure 10:**
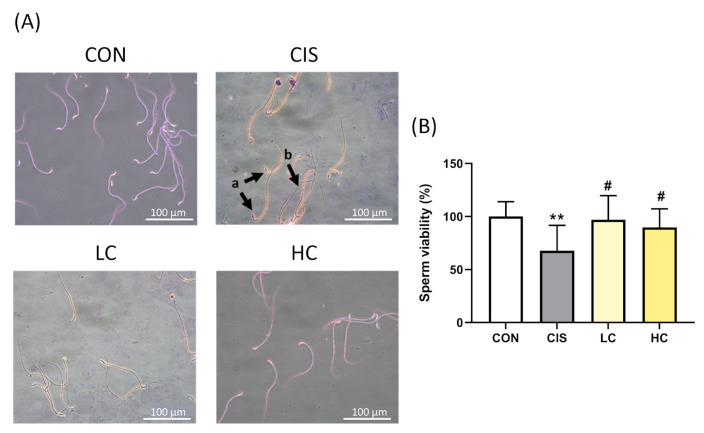
Effect of L-cysteine (CYS) on sperm morphology and viability in cisplatin (CIS)-induced animal model. (**A**) Sperm morphology stained with eosin Y and crystal violet, observed at 400× magnification. Black arrows indicate abnormal sperm, including (a) bent neck and (b) bent tail. (**B**) Sperm viability was assessed by counting the number of live and dead sperm. Data are represented as mean ± SEM. Different groups were analyzed using Student’s *t*-test. ** *p* < 0.01 compared with the CON group. # *p* < 0.05 compared with the CIS group. CON: control; CIS: cisplatin (10 mg/kg bw); LC: low CYS (100 mg/kg bw); and HC: high CYS (300 mg/kg bw).

Histological observations show the potential effectiveness of CYS in spermatogenesis. Sperm viability was measured by counting dead and live sperm. CYS treatment significantly reversed the harmful effects caused by CIS (Figure 10A). Sperm abnormalities were classified as abnormal head, midpiece, and tail using modified Eosin Y and crystal violet staining. The results showed that the sperm morphology in the CIS group had significantly more bent hooks and tails compared to the control group, resulting in reduced sperm viability. In contrast, CYS treatment improved the abnormal sperm morphology compared to the CIS group (Figure 10B) (CIS group: 67.75 ± 8.43 (*p* value = 0.004 compared with CON group (100.2 ± 4.38; LC group: 97.00 ± 7.19 (*p* value = 0.017 compared with CIS group); HC group: 89.78 ± 5.84 (*p* value = 0.04 compared with CIS group)). These results indicate that CYS can attenuate abnormal sperm morphology induced by CIS.

## 4. Discussion

Infertility is a global issue concerning human reproduction and is associated with environmental stress. Several factors could cause male infertility and they can be classified as congenital and acquired types [3]. Previous studies have suggested that a wide variety of endogenous and exogenous factors are known to perturb male reproductive function [33]. The side effects of certain drugs and chemicals also lead to testicular alteration. Although chemotherapy significantly targets dividing cancerous cells and improves survival rates in patients, the cytotoxic chemotherapeutic drug cisplatin not only kills tumor cells, but also causes a degree of damage to normal cells [34]. Malignant diseases might influence gonadal function through hormonal disorders and metabolic abnormalities. Cancer treatment has the potential to cause germ cell mutations that might increase the risk of growth disturbances and future infertility [35]. In addition to reproductive toxicity, alkylating agents are often limited due to adverse effects such as nephrotoxicity, ototoxicity, neurotoxicity, cardiotoxicity, hepatotoxicity, and gastrointestinal toxicity [36]. In the present study, the cisplatin-induced rat model showed that the SGOT, BUN, creatinine, and LDH levels in the serum were significantly increased compared the control group.

In this study, the highest dose used was 300 mg/kg. This is lower than the toxic doses reported, where 1000 to 2000 mg/kg/day resulted in significant adverse effects, including sperm granuloma, gastric erosion, salivation, and renal failure in some cases. The observed no-observed-adverse-effect levels (NOAELs) were less than 500 mg/kg/day for L-cysteine [37]. The conversion to a human clinical dosage is approximately 48.4 mg/kg/day [38]. Although this falls within a range used in some clinical settings, further studies are needed to confirm safety, particularly regarding long-term exposure and potential cumulative effects. The findings suggest that while 300 mg/kg is below the toxic threshold observed in this study, translating this dose to human use requires careful assessment of dose–response relationships and safety margins.

CIS is usually accompanied by numerous toxicities by binding to the N7 reactive center on purine residues of DNA, targeting DNA crosslinks and adducts. CIS administration causes DNA damage and initiates apoptotic or non-apoptotic cell death [39]. CIS-induced testicular toxicity’s underlying mechanism involves homeostatic disturbances resulting from oxidative stress and the formation of reactive oxygen species (ROS). However, under normal conditions, the body’s antioxidant capacity inhibits the accumulation of oxidative stress. When a condition is an imbalance between the production of ROS and body’s antioxidant system, these problems can threaten the health of testicular functions [40]. In particular, CIS induces mitochondrial oxidative stress, which impedes cholesterol transport to the inner mitochondrial membrane and disrupts steroidogenesis. The disturbance in mitochondrial function results in decreased expression of P450scc, a key enzyme in testosterone biosynthesis, leading to impaired testosterone production and diminished reproductive capacity [41]. Consistent with the present study, the results indicate that the primary cause of testicular cell damage is due to excessive ROS production, which is accompanied by mitochondrial oxidative stress and P450scc reduction. These findings highlight the central role of mitochondrial oxidative stress in mediating CIS-induced testicular toxicity and underscore the potential for targeted antioxidant therapies to mitigate these effects.

For the treatment of reproductive abnormality, other than surgery and hormone replacement/medications, natural product research is widely considered a powerful method for safe and convenient medications. Several studies have reported that natural compounds with antioxidant properties can fight reproductive threats, including L-carnitine [42], vitamin C/E [20,43,44,45,46], Lycopene [47,48,49], Melatonin [50,51] and Resveratrol [52,53], etc. This study mentioned that CYS is a sulfur-containing compound and is important for metabolic functions. N-Acetyl-l-cysteine (NAC) acts as cysteine metabolite and glutathione (GSH) precursor. In previous in vitro studies, NAC has been shown to regulate oxidant-induced cell damage by apoptosis [54]. In a Pulmonary fibrotic phenotype lung epithelial cell model, NAC eliminated fructose-induced mitochondrial ROS in A549 cells [55]. Then, administration of NAC also ameliorated LPS-induced inflammatory injury in in vivo and vitro models, reversing the change in HO-1 and NQO-1 mRNA levels, decreasing malondialdehyde (MDA) levels, and promoting antioxidant activities (CAT and SOD) in the lungs [56]. Another cisplatin induction study suggests that pretreatment with NAC restored GSH and SOD levels and reduced the MDA concentration in HepG2 cells [57]. Moreover, 20 mM NAC significantly decreased the fluorescence intensity of mitochondrial ROS in cisplatin-induced inner hair cells [58]. In agreement with our studies, these results showed that NAC and CYS are effective free radical scavengers and reduce ROS production in cisplatin-induced cells.

Previously, it has been reported that there are side effects of the ROS generated by the cisplatin drug, which indirectly depletes the concentration of reduced glutathione and promotes the imbalance of antioxidants. Further, it causes irreversible damage such as lipid oxidation and DNA repair failure and contributes to cytotoxicity and apoptosis. The research indicates that there are two main apoptotic pathways: the extrinsic or death receptor pathway and the intrinsic or mitochondrial pathway [59]. Intrinsic regulation of these apoptotic mitochondrial events occurs through members of the Bcl-2 family of proteins [60]. Release of Cytochrome C (Cyt C) from mitochondria is considered a key initial step in the apoptotic process. B-cell lymphoma protein 2 (Bcl-2)-associated X (Bax) protein activates the cascade of reactions by releasing Cyt C and downregulates Bcl-2 protein, which triggers successive activation of caspases and leads to cell death [61]. Wang W et al. showed that the expression of Caspase 3 and the number of TUNEL-positive inner hair cells were reduced in CIS (15–50 mM) induced after NAC intervention [58]. In an in vivo study, a single dose of 7.5 mg/kg to a cisplatin rat model showed that Caspase 3 expression in testicular cells was improved in NAC-treated rats [62]. Similarly, our study found that CYS lowers apoptotic rates in vitro and reduces PARP protein expression in the testis in vivo experiments.

High levels of ROS, as signaling molecules, has a key role in the inflammatory process and may result in cell and tissue injury. Then, inflammation would lead to increased levels of reactive oxygen species (ROS) [63,64]. It could be said that oxidative stress and inflammation have a complex relationship and vicious cycles. The use of CIS in cancer therapy is usually accompanied by inflammation [65] and increased pro-inflammatory cytokines like tumor necrosis factor-alpha (TNF-α), inducible nitric oxide synthase (iNOS), interleukin-1 beta (IL-1β), and Interleukin-6 (IL-6) [28,66,67]. On the other hand, research on matrine (MAT), which plays multiple pharmacological roles, found that it protected against cisplatin-induced acute kidney injury (AKI) by antioxidative stress and anti-inflammation actions via the SIRT3/OPA1 pathway [68]. MAT’s effect is similar to our antioxidant CYS effect; our results showed that CYS inhibits the protein expression of inflammatory response in TM3 cells.

The present study showed that TM4 Sertoli cells, as native Sertoli cells, aid germ cells in their development, proliferation, and maturation during spermatogenesis. The mature Sertoli cells form a complex network of specific intercellular junctions, which is called blood–testis barrier (BTB) structure. The junction markers, including ZO-1, Occludin, Claudin, and Connexin 43 (Cx43), prevent toxins entering the seminiferous tubules [27]. Several studies have demonstrated the adverse effects of chemotherapeutic agents’ toxicity on junctional intercellular communication [69,70]. Therefore, our results suggest that CYS enhances ZO-1 protein expression, which recovers the completeness of BTB structure.

Futher, a previous study reported that histological evaluation of cisplatin-treated testes showed disrupted testicular architecture, degenerative changes, and necrosis of spermatic cells. These apoptotic cells developed in the testis. CYS or Honokiol could recover testicular histology [71]. Then, computer-assisted sperm analysis (CASA) and acrosome reaction assay results showed that Honokiol improved sperm count and motility and reduced the damage to acrosome reaction ability [72]. Consistent with our vivo study, CYS slightly reduces the abnormal rate of sperm morphology and significantly elevates testicular structure integrity and spermatogenesis.

Several clinical trials have enabled identification of the effects of the usage of L-cysteine in human health and wellness. CYS analogs ameliorated cisplatin-induced reproductive toxicity. It was indicated that D-Ribose-l-Cysteine improved sperm parameters and increased serum testosterone, FSH, LH, and testicular 17β-HSD activity because of the increase in antioxidant capacity. Therefore, D-Ribose-l-Cysteine had positive effects on spermatogenesis and maintained testicular tissues [73]. This highlights the potential application of CYS in treating reproductive disorders.

One limitation of our study is the use of cell lines rather than tissue sections to evaluate BTB-related proteins. While the cell model enabled targeted analysis of specific cell types (Leydig and Sertoli cells), it may not fully represent the BTB’s structural and functional complexity, as seen in whole tissue. Future studies should consider tissue-based approaches, including IHC or IF, to provide a more in-depth understanding of BTB integrity in response to cisplatin exposure. Due to equipment constraints, we were only able to perform viability assessments of sperm, focusing on motile and non-motile sperm as indicators of functional changes post-treatment. Although these measures are commonly used to assess sperm quality and correlate with fertility potential [74], a more detailed analysis of sperm populations, including sperm morphology and additional subpopulations, would have provided a more comprehensive characterization of the effects of CYS on sperm health.

## 5. Conclusions

Our findings reveal that cisplatin inflicts substantial damage on testicular cells, leading to apoptosis, inflammation, and impaired survival of TM3 and TM4 cells. Treatment with L-cysteine demonstrated a protective capacity, not only by reducing cellular apoptosis and inflammation but also by alleviating mitochondrial oxidative stress and restoring testosterone secretion in vitro. In vivo, L-cysteine pre-treatment combined with cotreatment markedly improved sperm viability and restored serum testosterone levels. Furthermore, the elevation in CYP11A1 protein expression underscores L-cysteine’s role in modulating steroidogenesis, facilitating spermatogenic recovery, and reinforcing reproductive function. Together, these data position L-cysteine as a promising agent for countering cisplatin-induced testicular and spermatogenic impairment, underscoring its therapeutic potential for fertility preservation in chemotherapy contexts.

## Data Availability

The data supporting the findings of this study are available from the corresponding author upon reasonable request.
